# Feasibility, Acceptability, and Preliminary Efficacy of a Smartphone App–Led Cognitive Behavioral Therapy for Depression Under Therapist Supervision: Open Trial

**DOI:** 10.2196/53998

**Published:** 2024-04-09

**Authors:** Sabine Wilhelm, Emily E Bernstein, Kate H Bentley, Ivar Snorrason, Susanne S Hoeppner, Dalton Klare, Jennifer L Greenberg, Hilary Weingarden, Thomas H McCoy, Oliver Harrison

**Affiliations:** 1 Massachusetts General Hospital, Harvard Medical School Boston, MA United States; 2 Koa Health London United Kingdom

**Keywords:** depressive disorder, depressive, depression, open trial, open trials, single arm, smartphone, cognitive behavioral therapy, cognitive behavioural therapy, CBT, psychotherapy, psychoeducation, digital health, mobile applications, mHealth, mobile health, app, apps, application, applications, psychiatry, psychiatric, feasibility, acceptability, usability, satisfaction, user experience, mental

## Abstract

**Background:**

Major depressive disorder affects approximately 1 in 5 adults during their lifetime and is the leading cause of disability worldwide. Yet, a minority receive adequate treatment due to person-level (eg, geographical distance to providers) and systems-level (eg, shortage of trained providers) barriers. Digital tools could improve this treatment gap by reducing the time and frequency of therapy sessions needed for effective treatment through the provision of flexible, automated support.

**Objective:**

This study aimed to examine the feasibility, acceptability, and preliminary clinical effect of Mindset for Depression, a deployment-ready 8-week smartphone-based cognitive behavioral therapy (CBT) supported by brief teletherapy appointments with a therapist.

**Methods:**

This 8-week, single-arm open trial tested the Mindset for Depression app when combined with 8 brief (16-25 minutes) video conferencing visits with a licensed doctoral-level CBT therapist (n=28 participants). The app offers flexible, accessible psychoeducation, CBT skills practice, and support to patients as well as clinician guidance to promote sustained engagement, monitor safety, and tailor treatment to individual patient needs. To increase accessibility and thus generalizability, all study procedures were conducted remotely. Feasibility and acceptability were assessed via attrition, patient expectations and feedback, and treatment utilization. The primary clinical outcome measure was the clinician-rated Hamilton Depression Rating Scale, administered at pretreatment, midpoint, and posttreatment. Secondary measures of functional impairment and quality of life as well as maintenance of gains (3-month follow-up) were also collected.

**Results:**

Treatment credibility (week 4), expectancy (week 4), and satisfaction (week 8) were moderate to high, and attrition was low (n=2, 7%). Participants self-reported using the app or practicing (either on or off the app) the CBT skills taught in the app for a median of 50 (IQR 30-60; week 4) or 60 (IQR 30-90; week 8) minutes per week; participants accessed the app on an average 36.8 (SD 10.0) days and completed a median of 7 of 8 (IQR 6-8) steps by the week 8 assessment. The app was rated positively across domains of engagement, functionality, aesthetics, and information. Participants’ depression severity scores decreased from an average Hamilton Depression Rating Scale score indicating moderate depression (mean 19.1, SD 5.0) at baseline to a week 8 mean score indicating mild depression (mean 10.8, SD 6.1; *d=*1.47; *P*<.001). Improvement was also observed for functional impairment and quality of life. Gains were maintained at 3-month follow-up.

**Conclusions:**

The results show that Mindset for Depression is a feasible and acceptable treatment option for individuals with major depressive disorder. This smartphone-led treatment holds promise to be an efficacious, scalable, and cost-effective treatment option. The next steps include testing Mindset for Depression in a fully powered randomized controlled trial and real-world clinical settings.

**Trial Registration:**

ClinicalTrials.gov NCT05386329; https://clinicaltrials.gov/study/NCT05386329?term=NCT05386329

## Introduction

Major depressive disorder (MDD), characterized by hallmark symptoms of persistent depressed mood and loss of interest in activities [1], is highly prevalent. In 2020, an estimated 21 million adults in the United States were impacted (8.4% population prevalence) [2]. The rates of elevated depressive symptoms have continued to rise since the COVID-19 pandemic, now affecting nearly 1 in 3 adults [3]. Depression is the leading cause of disability worldwide [4] and is associated with economic costs exceeding US $326.2 billion in the United States alone [5]. Despite the substantial personal and societal impact of MDD, a minority of individuals meeting diagnostic criteria—let alone those at risk or with subthreshold symptoms—receive care; even fewer receive minimally adequate treatment (estimates range from 3% in low- to middle-income countries and 23% in high-income countries [6]), such as cognitive behavioral therapy (CBT), the most widely studied and recommended psychotherapy [7]. At a systems level, the limited availability of trained clinicians is a substantial contributor to low treatment utilization [8]. There simply are not and will not be enough clinicians to meet current demands for mental health care. Additionally, many people do not seek treatment for depression due to obstacles such as geographic distance from care providers, high costs, and stigma [8,9]. Given the prevalence of MDD and the substantial treatment gaps that exist, there is a clear need for low barrier, more widely accessible, effective treatments for MDD.

The technology could bridge such gaps. Smartphone apps or other digital tools could reduce the time and frequency of sessions by supplementing clinician effort with automated, validated support that can be used flexibly between sessions [10,11]. However, standalone apps are not sufficient or appealing for many patients [12,13]. The majority of apps, even those that are grounded in empirically supported treatments, have high dropout rates, which limits their effectiveness [14-16]. The absence of concurrent human support is often cited as the major reason for nonadherence or nonengagement [13,17-19]. Some engagement is likely a minimum requirement for an app-based therapy to be effective; guidance from a trained provider should further mitigate issues of comprehension, personalization, problem-solving, and interference from comorbid or life concerns [20]. Equivalent effects of face-to-face CBT and internet-delivered CBT for depression have been found for treatments that are *therapist guided*, meaning patients are in contact with a therapist throughout treatment (eg, weekly sessions, check-in phone calls, asynchronous, messaging, and weekly feedback emails) [21-23]*.* Moreover, many users simply want access to a therapist and are less willing to engage in self-directed digital treatments [24,25]. Thus, a digital service that combines mobile-based CBT with brief remote individual sessions with a clinician (ie, teletherapy monitoring) has the potential to greatly enhance the scalability of high-quality app-based treatment, particularly for moderately and severely ill patients while reducing clinician burden and cost [26-28].

The purpose of this study was to conduct an open trial to test the feasibility, acceptability, and efficacy of the Mindset for Depression app (a novel, smartphone-based CBT program) with brief video-conferencing appointments with a therapist. We hypothesized that the treatment would be feasible and acceptable. We also hypothesized that treatment would yield statistically significant reductions in depression symptom severity (primary clinical outcome) as well as improvements in functioning and quality of life (secondary clinical outcomes) from baseline to posttreatment (week 8). The treatment was tested for patients with moderate to severe depression: those who would typically be referred for one-on-one outpatient therapy [29].

## Methods

### Study Design

This open trial tested the Mindset for Depression app when combined with brief (16-25 minutes) video-conferencing visits with a CBT therapist over 8 weeks. The primary outcomes were feasibility, acceptability, and preliminary efficacy, as measured by change in depression symptom severity. To increase accessibility and thus generalizability, all study procedures were conducted remotely.

### Ethical Considerations

The study was approved by the institutional review board of Massachusetts General Hospital (2020P001958). All participants provided informed consent prior to the initiation of study procedures and were given the ability to opt out at any point. Data were deidentified to protect participants’ privacy. Participants were compensated US $25 at mid-treatment, end of treatment, and 3-month follow-up assessments.

### Participants

Eligible participants, recruited between May 2022 and February 2023, were at least 18 years old, living in Massachusetts, presenting with a current primary *Diagnostic and Statistical Manual of Mental Disorders: 5th Edition* (*DSM-5*) diagnosis of MDD, and experiencing at least moderately severe symptoms (Patient Health Questionnaire-9 [PHQ-9] score ≥ 10). Participants taking psychotropic medication were on a stable dose for at least 2 months prior to enrollment and were asked to remain on the same stable dose throughout the study period. Exclusion criteria included 4 or more prior sessions of CBT for depression (assessed via self-report and interview with an independent evaluator), current severe substance use disorder, lifetime bipolar disorder or psychosis, acute and active suicidal ideation as indicated by clinical judgment, a score ≥ 2 on the past month suicidal ideation subscale of the Columbia-Suicide Severity Rating Scale [30], concurrent psychological treatment, and inability to engage with treatment (eg, did not own a supported smartphone).

### Procedure

#### Treatment

The Mindset for Depression app provides key CBT-derived content for adults with MDD and was designed to be used in conjunction with a therapist over 8 weeks. The duration of CBT trials typically ranges from 6 to 20 sessions [31]. Mindset for Depression was built in collaboration between researchers at the Massachusetts General Hospital and Koa Health. The app-based format allows participants to review CBT content and accompanying skills practice exercises at their convenience and own pace and with support from their therapist.

The app and clinician dashboard were developed through collaborative, user-centered design, integrating perspectives from clinicians (MDs and psychologists with expertise in MDD and CBT), digital health researchers, patients with MDD and experience with CBT and other therapies, engineers, and designers. Through this approach, the product being tested was deployment ready (eg, built on a commercial platform, able to be quickly scaled and professionally maintained to minimize technical difficulties, and ensure compliance with up-to-date privacy and security standards) and therefore well positioned to succeed outside of research studies [32].

#### CBT Modules

**Figure 1:**
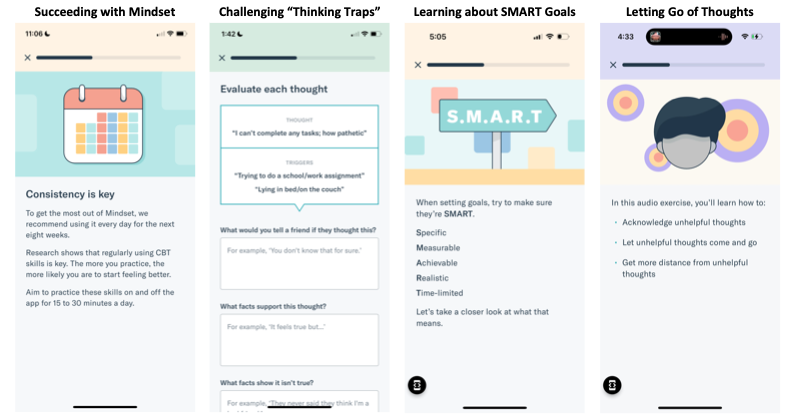
Screenshots from the Mindset for Depression smartphone app. CBT: cognitive behavioral therapy; SMART: specific, measurable, achievable, relevant, and time-bound.

The app delivers content in 8 steps, corresponding to the 8 weeks of treatment. A summary of these steps is visualized in Table 1. Participants were also allowed access to the app during the 3-month follow-up period. Core CBT skills included across treatment include psychoeducation, cognitive restructuring and core beliefs, behavioral activation, mindfulness, and relapse prevention [33,34]. Step 1 comprises psychoeducation about MDD and the CBT model and background and skills practice for identifying and restructuring “thinking traps,” or maladaptive automatic thoughts [35,36]. Step 2 focuses on the short- and long-term impact of withdrawal and avoidance on mood and provides a structure for recording daily activities and monitoring associated moods. Step 3 introduces behavioral activation and scheduling and provides guidance for identifying valued or new activities and setting specific, measurable, achievable, relevant, and time-bound (SMART) goals [37,38]. Activity scheduling and monitoring (ie, recording completed activities and associated mood ratings), with an emphasis on personal values and meaning, continue for the remainder of treatment. Step 4 introduces mindfulness (present-focused and nonjudgmental awareness) and offers a guided mindful breathing audio exercise [39]. Steps 5 and 6 provide users with additional mindfulness approaches, including grounding and letting go of unhelpful thoughts. Step 7 builds on prior cognitive skills and delves into the definition of core beliefs, their relationship to automatic thoughts and feelings, and strategies to identify and challenge them (eg, downward arrow technique and building self-esteem). Step 8 concludes with relapse prevention by helping users consolidate treatment skills, anticipate future challenges, and plan for continued practice and flexible use of skills. Example screenshots from the smartphone app are included in Figure 1.

**Table 1:** Summary of steps in the Mindset for Depression program.

	Step 1	Step 2	Step 3	Step 4	Step 5	Step 6	Step 7	Step 8
Psychoeducation	✓							
Cognitive restructuring	✓							
Relationship between behavior and mood		✓						
Behavioral activation			✓	✓	✓	✓	✓	✓
Mindfulness				✓	✓	✓		
Modifying core beliefs and building self-esteem							✓	
Relapse prevention								✓

#### Therapists

Each participant was matched with a licensed doctoral-level therapist. Therapists were trained in and actively practicing CBT for MDD and provided with study-specific training in using the Mindset app and therapist dashboard prior to beginning the trial. To ensure proficiency, therapists were required to complete the Massachusetts General Hospital Psychiatry Academy CBT training course and pass (>90% correct) both the corresponding CBT knowledge test and an MDD knowledge test. Weekly supervision from the principal investigator (expert in CBT) was also provided. To ensure ongoing high-quality treatment, including that implementation fidelity targets were met and non-CBT techniques were absent, sessions were audio recorded, and 40 of the 224 planned sessions (18.9% of the 212 sessions ultimately conducted) were randomly selected and rated for competency and treatment adherence by an independent rater. Adherence raters were experienced in CBT for MDD and further trained and supervised. Core elements of each treatment session (5-6 items) were rated for adherence on a 7-point scale (1=not at all to 7=completely adherent) and then a global rating of adherence was assigned. The full adherence scale is included in Multimedia Appendix 1. Competence was rated on 12 aspects (32 items) of CBT for MDD (eg, positive outlook, knowledge, clear communication, empathy, flexibility, and empowering the patient). Each item was scored on a 5-point scale (1=not at all to 5=completely competent) and then a global rating of competence was assigned. Overall, adherence and competence were high, with 100% of all rated sessions evaluated as “completely” adherent and 100% of all rated sessions evaluated as “mostly” or “completely” competent.

Therapists offered each patient 8 video-conferencing appointments (16-25 minutes; via HIPAA [Health Insurance Portability and Accountability Act]–compliant video conference) to be conducted weekly. This duration of appointment corresponds to a clinician billing code (CPT-90832), helping to ensure that the reimbursement of clinician time would not become a barrier to scale-up following the research. As needed, because of a therapist’s or patient’s schedule, up to 2 sessions were able to be scheduled per week. Throughout the treatment, participants were able to communicate with their therapists between sessions through asynchronous in-app secure messaging. Sessions were meant to support a patient’s progress through the app-led treatment. In this way, the model mimicked the “flipped classroom,” a new pedagogical approach shown to improve student learning [40]. In a flipped classroom, students watch or read lectures and complete initial practice problems asynchronously, reserving valuable classroom time for active problem-solving with an instructor. As such, sessions were intended to monitor risk as needed, help participants set goals, enhance motivation, clarify and practice the skills learned via the Mindset app to best meet the patient’s needs, brainstorm ideas for homework, and problem-solve treatment barriers that arose. Therapists were instructed to work within a CBT framework and not to introduce other treatment modalities. Such fidelity was monitored in weekly supervision, via therapist self-checks included within session records (“Did you use any of the following non-CBT techniques? [check *all* that apply]”) and via adherence ratings (ie, the degree to which forbidden content was introduced). The therapist dashboard was a separate web-based portal wherein therapists could receive and respond to messages and track participant progress in the app.

#### Assessments

Assessments were conducted by master’s or doctoral-level independent evaluators who were not involved in treatment, were complemented by participant self-report, and occurred at baseline, mid-treatment (week 4), end of treatment (week 8), and follow-up (3 months posttreatment). Evaluators completed training on all clinician-administered measures and were required to maintain high reliability (>0.75 intraclass correlation coefficient), with a gold standard expert rater; 18.9% (20/106) of randomly selected assessments were rated to prevent rater drift. Evaluators were not privy to participants’ progress in treatment (eg, app content reviewed and session notes). Adverse events, life events, and changes in medication or outside treatment were surveyed at each assessment or when a patient reported to study staff.

### Measures Descriptions

#### Baseline Diagnostic Assessment

The Mini International Neuropsychiatric Interview was used to establish eligibility and characterize the sample. It is a reliable, validated semistructured diagnostic assessment of *DSM-5* psychiatric disorders [41].

#### Feasibility and Acceptability

Participants completed the self-reported measures as follows. The Credibility/Expectancy Questionnaire (CEQ) [42], completed at baseline and week 4, is a 6-item, self-reported Likert-type questionnaire that assesses patients’ judgments about the credibility of the treatment rationale and treatment expectancy. Items on both subscales are summed together for total outcome scores that can range from 3 to 27, where higher scores mean higher treatment credibility and higher outcome expectancy. We assessed the internal consistency of scales with coefficient omega (McDonald ω), given the heterogeneity of variances across scale items; coefficient ω can be interpreted in the same way as Cronbach α. The internal consistency of the credibility items in this sample ranged from ω=0.69 at baseline to ω=0.77 at week 4; for the expectancy items, internal consistency ranged from ω=0.82 at baseline to ω=0.96 at week 4. The Mobile Application Rating Scale User Version (uMARS) [43], administered at week 8, collects evaluations of mobile health apps. The 26 items assess participants’ evaluations of engagement, functionality, aesthetics, information quality, app subjectivity quality, and perceived impact. Items are rated on differently worded 5-point Likert scales ranging from 1 (inadequate) to 5 (excellent). An overall app rating score can be calculated as the mean score of the first 4 subscales (engagement, functionality, aesthetics, and information quality; a range of 1-5), where higher scores indicate higher overall perceived app quality. In this sample, the internal consistency of the 4 subscales used in the overall mean scores were ω=0.76 for engagement, ω=0.83 for functionality, ω=0.72 for aesthetics, and ω=0.62 for information quality, with an overall item consistency of ω=0.83. The Client Satisfaction Questionnaire (CSQ) [44], completed at weeks 4 and 8, is an 8-item questionnaire assessing satisfaction with clinical services received. Each item uses a 4-point Likert scale. Items are summed for a total score ranging from 8 to 32, with higher scores indicating greater satisfaction. The internal consistency of the CSQ was ω=0.93 at week 4 and ω=0.89 at week 8. Treatment use was assessed with a single question: “On average, how much time (in minutes) do you spend using the app or practicing skills from the app in total, per week?” Answers were collected as the number of minutes in integer format, where more time spent on and off the app was interpreted as greater treatment use. In addition, app use data were collected automatically based on the actions participants completed in the app. Due to technical issues, 2 participants’ app use data were inadvertently not recorded. The internal consistency values for the CEQ credibility subscale at baseline and the uMARS information quality subscale fell below 0.7 and are a noted limitation.

#### Clinician-Administered Measures

The primary measure of MDD symptom severity was the (clinician-rated) Hamilton Depression Rating Scale (HAM-D) [45]. Considered a gold standard means of assessing symptom severity in patients who are depressed, it contains 21 items that are rated on a mixture of 3- and 5-point Likert scales. The first 17 items are summed for the total score, which can range from 0 to 52. Higher scores indicate greater depression severity. The internal consistency of the HAM-D in this sample ranged from ω=0.79 at baseline to ω=0.93 at the 3-month follow-up (question 17 was necessarily omitted from internal consistency calculations due to the absence of variability in responses; all participants received a score of 0 for this “insight” item [“Acknowledges being depressed and ill”] at all assessment points with the exception of 1 participant at the 3-month follow-up). To evaluate treatment response and remission, we used criteria of HAM-D score reductions of ≥50% for treatment response, HAM-D score reductions of ≥25% but < 50% for partial response, and HAM-D scores ≤7 to indicate remission [46-48]. An expert rater reviewed 18.9% (20 of the 106 assessments that were ultimately completed) of HAM-D assessments. Inter-rater reliability was excellent (HAM-D: intraclass correlation coefficient (1,1)=0.91).

#### Self-Reported Measures

Participants completed the following secondary measures of symptoms and functioning at each assessment: (1) The Work and Social Adjustment Scale (WSAS) [49] is a 5-item, self-reported measure of impairment in occupational, social, and family domains. Items are measured on 9-point Likert scales ranging from 0 (no impairment at all) to 8 (very severe impairment). The items are summed for a total score ranging from 0 to 40, where higher scores mean higher functional impairment. The internal consistency of the WSAS ranged from ω=0.84 at baseline to ω=0.93 at the 3-month follow-up. (2) The Quality of Life Enjoyment and Satisfaction Questionnaire-Short Form (Q-LES-Q-SF) [50] is a 16-item self-reported measure of subjective quality of life. Each question is rated on a 5-point Likert scale ranging from 1 (very poor) to 5 (very good). Questions 1-14 are then summed to a total score, and the total score is reported as a percentage maximum possible, such that the final percent score range is 0% to 100%; higher scores correspond to greater ratings of quality of life. The internal consistency of the Q-LES-Q-SF ranged from ω=0.84 at baseline to ω=0.90 at the 3-month follow-up. (3) The PHQ-9 [51] is a self-reported measure of the past week’s depression severity. It includes 9 Likert scale items mapping onto *DSM-5* symptom criteria and ranging from 0 (not at all) to 3 (every day). The internal consistency of the PHQ-9 ranged from ω=0.70 at baseline to ω=0.89 at week 8.

### Data Analysis

#### Power Analysis

With 28 participants enrolled, we had > 80% power to detect pre- to posttreatment effect sizes of *d* ≥1.37 (very large effect sizes), assuming 30% dropout, a pre- to posttreatment correlation of 0.18, and a doubling of the SD from pre- to posttreatment. The pre- to posttreatment correlation estimate was based on the mean pooled correlation between pretest and posttest HAM-D scores in 14 CBT trials for adult depression [52], and the estimate of the detec effect size was based on a single degree of freedom contrast in a paired means test implemented in SAS for Windows (version 9.4; SAS Institute).

#### Feasibility and Acceptability

We examined feasibility and acceptability by reporting (1) dropout rates and reasons (defined as participants not completing an end point HAM-D), (2) patient satisfaction (CSQ), (3) patient feedback (uMARS), (4) patient credibility and expectancy ratings (CEQ), and (5) treatment use. We computed means and SDs for the number of app steps completed, the number of days on which participants completed any actions in the app, the number of messages participants sent their therapist in the app, the number of sessions participants completed with their therapists, and session time spent per patient per week by therapists. For measures collected at least twice (ie, CSQ, CEQ credibility and expectancy, and treatment use), we used generalized linear mixed models with repeated measures to examine if these self-reported ratings changed over time.

#### Preliminary Efficacy and Secondary Outcomes

Analyses were first completed using our intent-to-treat sample (participants who completed a baseline assessment) and then repeated with our “per-protocol” sample (participants who completed posttreatment assessments and did not change psychiatric medications or begin psychotherapy during the study; n=24, 86%). We examined the preliminary efficacy of Mindset for Depression plus brief video-conferencing appointments with a therapist on symptoms and well-being outcomes using mixed model analyses with repeated measures (baseline, mid-treatment, posttreatment, and 3-month follow-up) modeled using an unstructured covariance matrix. We then compared pre- to posttreatment differences using a 2-tailed α of .05 to evaluate preliminary efficacy. We similarly compared pretreatment to end of follow-up estimates to estimate whether changes remained significant by the end of the follow-up. Means are presented as raw means with SDs, while differences between assessments are presented as model-estimated means with CIs (LSM differences [95% CI]) unless otherwise specified. Effect sizes were calculated as Hedges *g_ave_*, which takes the correlation of within-participant scores into account [53]. Analyses were conducted for changes in depression symptoms (HAM-D and PHQ-9), functional impairment (WSAS), and quality of life (Q-LES-Q-SF). All analyses were completed using the SAS software (version 9.4, SAS Institute Inc).

## Results

### Overview

Study participants (N=28) were predominantly female (n=21, 75%), White (n=20, 71%), and single (n=19, 70%), with a mean age of 33.5 (SD 10.9) years. The majority of participants had college or advanced degrees, were employed full time, and came from urban or suburban locations (Tables 2 and 3). Nearly half of the participants (n=13, 45%) had one or more comorbid psychiatric diagnoses, and the average duration of MDD was 15.5 (SD 12.7) years.

**Table 2:** Baseline demographics of participants enrolled in the Mindset open trial.

Demographics		Values
Age (years), mean (SD)		33.5 (10.9)
**Sex at birth, n (%)**		
	Female	75 (21)
	Male	25 (7)
**Gender identity, n (%)**		
	Women	75 (21)
	Men	25 (7)
**Sexual orientation, n (%)**		
	Straight or heterosexual	71 (20)
	Bisexual	18 (5)
	Lesbian, gay, or homosexual	4 (1)
	Other	7 (2)
Hispanic ethnicity, n (%)		18 (5)
**Race, n (%)**		
	Asian or Pacific Islander	11 (3)
	Black	4 (1)
	White	71 (20)
	Other	14 (4)
**Education, n (%)**		
	Less than or equal to a high school graduate	14 (4)
	Technical school or some college	18 (5)
	College graduate	39 (11)
	Graduate or professional school	29 (8)
**Marital status, n (%)**		
	Single, never married	68 (19)
	Married	18 (5)
	Partnered	7 (2)
	Separated or widowed	7 (2)
**Employment, n (%)**		
	Full time (≥35 hours per week)	86 (24)
	Student	7 (2)
	Unemployed	4 (1)
	Retired	4 (1)
**Household income (US $), n (%)**		
	$34,999 or less	11 (3)
	$35,000-74,999	25 (7)
	$75,000-149,999	50 (14)
	$150,000 or more	14 (4)
**Geographic location, n (%)**		
	Urban	43 (12)
	Suburban	46 (13)
	Rural	11 (3)

**Table 3:** Baseline clinical characteristics of participants enrolled in the Mindset open trial.

Characteristics			Values
Duration of MDD^a^ (years), mean (SD)			15.5 (12.7)
**Current psychiatric comorbidities (*DSM-5*^b^ diagnoses)^c^, n (%)**			
	Agoraphobia	7 (2)	
	Alcohol use disorder	11 (3)	
	Generalized anxiety disorder	21 (6)	
	Social anxiety disorder	14 (4)	
	Other	18 (5)	
**Number of psychiatric comorbidities, n (%)**			
	None	54 (15)	
	1	36 (10)	
	2	0 (0)	
	3 or more	11 (3)	
**Current psychotropic medication^c^, n (%)**			
	None	57 (16)	
	SRI^d^	21 (6)	
	Non-SRI antidepressant	18 (5)	
	Other psychotropic medication^e^	14 (4)	

^a^MDD: major depressive disorder.

bDSM-5: Diagnostic and Statistical Manual of Mental Disorders, Fifth Edition.

^c^Percentage sums may exceed 100% because participants could report more than one diagnosis or be on more than 1 s psychotropic medication.

^d^SRI: serotonin reuptake inhibitor.

^e^This included anticonvulsants; no participant reported taking antipsychotics.

#### Feasibility and Acceptability

**Figure 2:**
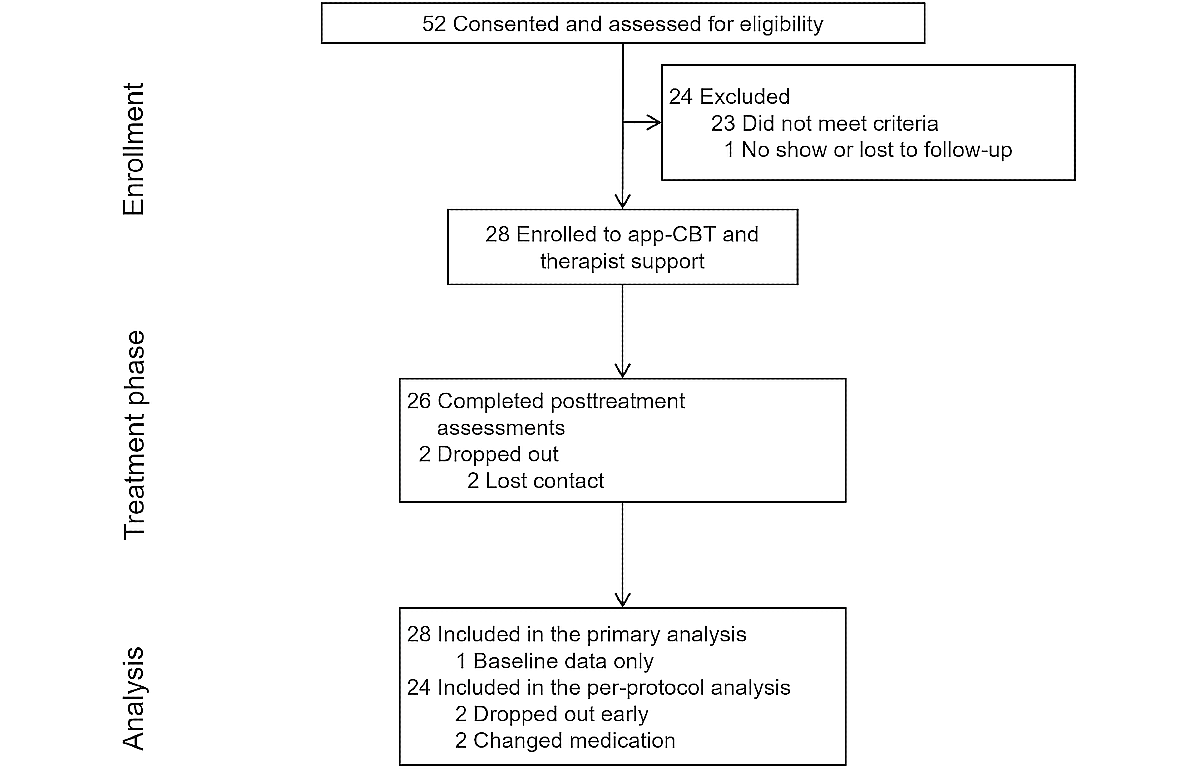
Flow of participants through the 8-week open trial of the Mindset Depression app with brief therapist visits on the web for people with a primary diagnosis of major depressive disorder. Reasons for ineligibility include diagnosis of bipolar disorder or severe substance use disorder, PHQ-9 score < 10, past CBT for MDD, acute, active suicidal ideation, and MDD not being the primary diagnosis. CBT: cognitive behavioral therapy.

Credibility and expectancy scores were moderate to high at pre- and mid-treatment. The ratings did not differ significantly between timepoints. Mean credibility ratings were 18.9 (SD 3.1) at pretreatment and 19.3 (SD 3.8) at mid-treatment (LSM difference 0.4, 95% CI –1.4 to 2.2; *P*=.63; *g_ave_*=0.13). Mean expectancy ratings were 13.8 (SD 3.3) at pretreatment and 15.0 (SD 5.4) at mid-treatment (LSM difference 1.2, 95% CI –0.7-3.0; *P*=.22; *g_ave_*=0.28). Of the 28 participants, 2 (7%) dropped out of the study prior to their posttreatment assessment at week 8 (Figure 2): 1 during the first week of treatment and 1 after the midpoint assessment; both participants were lost to follow-up despite repeated contact attempts and neither provided a reason for drop out. One more participant was lost to follow-up after the posttreatment assessment. Among the 26 participants who completed the posttreatment assessment, patient satisfaction was high and did not change significantly from mid-treatment (CSQ total score mean 26.3, SD 4.0) to posttreatment (mean 27.2, SD 3.3; LSM difference 1.0, 95% CI –0.1 to 2.2; *P*=.07; *g_ave_*=0.24). Conservatively counting the 2 dropouts as not satisfied, 89% (16/28) were very or mostly (9/28) satisfied and 93% (26/28) would recommend the Mindset for Depression program.

With respect to app use and satisfaction, participants reported practicing skills from the app on their smartphone and offline for a median of 50 (IQR 30-60) minutes per week up to mid-treatment and 60 (IQR 30-90) minutes per week between mid- and posttreatment. Based on passively collected app use data (n=26), participants accessed the app on 36.8 (SD 10.0) days, completed a median of 7 (IQR 6-8) steps out of 8 steps by the week 8 assessment, and sent a median of 0 (IQR 0-4) between session messages to their therapist through the app. Five participants completed the last assigned step after the week 8 assessment, bringing step completion to a median of 8 (IQR 6-8) by the end of follow-up (3 months), with 58% (15/26) participants completing the final step by then. Participants’ overall ratings of the app quality, rated on the 1 (inadequate) to 5 (excellent) scale of the uMARS, was high (mean 4.3, SD 0.4); ratings of the app’s functionality (mean 4.5, SD 0.6), aesthetics (mean 4.6, SD 0.4), and information (mean 4.6, SD 0.4) were higher than those of the engagement subscale (mean 3.6, SD 0.6). Participants reported a mean overall star rating of 4.0 (SD 0.5) but were less inclined to endorse that they would be willing to pay for the app (mean 2.5, SD 1.2).

With respect to therapist support, participants attended an average of 7.6 (SD 1.5) of the possible 8 brief sessions, each of which lasted approximately 24.5 (SD 1.1) minutes. In the sessions, therapists mainly covered behavioral strategies (mean 10.9, SD 3.4 minutes), cognitive strategies (mean 6.9, SD 2.7 minutes), psychoeducation (mean 2.5, SD 2.9 minutes), and mindfulness strategies (mean 2.4, SD 1.2 minutes), with only a little time (<2 minutes on average) spent on explicit motivational strategies, risk management, and technical issues. Participants had a mean homework completion rate (per therapist report) of 82.7% (SD 13.8%).

#### Preliminary Efficacy and Secondary Outcomes

Over the course of the 8-week treatment, participants’ depression severity decreased significantly on both the clinician-rated (HAM-D: *P*<.001; *g_ave_*=1.47) and self-reported measures (PHQ-9: *P*<.001; *g_ave_*=1.89; 4). Concurrently, participants’ self-rated functional impairment decreased (WSAS: *P*<.001; g*_ave_*=1.29), and their self-rated quality of life increased (Q-LES-Q-SF: *P*<.001; *g_ave_*=1.74). These changes persisted through the 3-month follow-up, with effect sizes remaining largely the same (Table 4). The results did not differ meaningfully in the per-protocol analyses; HAM-D, PHQ-9, WSAS, and Q-LES-Q scores all improved with statistically significant and large effect sizes (Table 5).

Conservatively counting dropouts as having not responded to treatment, 46% (13/28) participants responded and another 7% (2/28) partially responded at posttreatment. Regarding remission, and again counting dropouts also as not remitting, 36% (10/28) achieved remission at posttreatment. Based on the per-protocol sample, 54% (13/24) participants fully responded to treatment and 8% (2/24) partially responded to treatment; 42% (10/24) achieved remission by the posttreatment assessment. By the end of follow-up and counting dropouts as not responding and not remitting, 50% (14/28) participants had responded to treatment, and an additional 21% (6/28) of participants had partially responded to treatment; 36% (10/28) participants were in remission. In the per-protocol sample at the end of follow-up and excluding the additional participant lost to follow-up, 61% (14/23) of participants had responded to treatment, and 26% (6/23) of participants had partially responded to treatment; 43% (10/23) participants were in remission at the 3-month follow-up assessment.

**Table 4:** Baseline, mid-treatment (week 4), end of treatment (week 8), and follow-up (week 20) estimated mean scores on key clinical outcome measures.

Outcome measure	Baseline, LSM^a^ (SE)	Week 8, LSM (SE)	Week 20, LSM (SE)	Estimated difference (week 0-8), LSM (95% CI)	*P* value	Effect size (week 0-8)^b^, Hedges g_av_	Estimated difference (week 0-20), LSM (95%CI)	*P* value	Effect size (week 0-20)^a^, Hedges *g*_*av*_
HAM-D^c^ total scores	19.1 (0.9)	11.3 (1.2)	10.4 (1.6)	–7.8 (–10.5 to –5.2)	<.001	1.47	–8.7 (–12.0 to –5.4)	<.001	1.44
PHQ-9-^d^ total scores	15.1 (0.7)	7.1 (1.0)	6.9 (0.9)	–8.0 (–9.9 to –6.1)	<.001	1.89	–8.2 (–10.2 to –6.3)	<.001	1.97
WSAS^e^ total scores	23.1 (1.5)	13.2 (1.5)	12.5 (1.8)	–9.9 (–13.5 to –6.4)	<.001	1.29	–10.6 (–14.5 to –6.8)	<.001	1.26
Q-LES-Q-SF^f^ % scores	40.9 (2.2)	62.5 (2.6)	62.8 (2.8)	21.5 (14.5 to 28.6)	<.001	1.74	21.9 (15.0 to 28.8)	<.001	1.73

^a^LSM: least squares mean.

^b^Within-group effect sizes were calculated as Hedges *g_ave_* for differences from baseline to week 8 or 20, respectively, using raw means data.

^c^HAM-D: Hamilton Depression Rating Scale (score range 0 to 52, where higher scores indicate greater depression severity).

^d^PHQ-9: Patient Health Questionnaire-9 item (score range 0 to 27, where higher scores indicate greater depression severity).

^e^WSAS: Work and Social Adjustment Scale (score range 0 to 40, where higher scores mean higher functional impairment).

^f^Q-LES-Q-SF: Quality of Life Enjoyment and Satisfaction Questionnaire- Short Form (percent score range 0% to 100%, where higher scores correspond to greater ratings of quality of life).

**Table 5:** Baseline, mid-treatment (week 4), end-of-treatment (week 8), and follow-up (week 20) estimated mean scores on key clinical outcome measures in the per-protocol sample (n=24).

Outcome measure	Baseline, LSM^a^ (SE)	Week 8, LSM (SE)	Week 20, LSM (SE)	Estimated difference (week 0-8), LSM (95% CI)	*P* value	Effect size (week 0-8)^b^, Hedges g_av_	Estimated difference (week 0-20), LSM (95% CI)	*P* value	Effect size (week 0-20)^a^, Hedges *g*_*av*_
HAM-D^c^ total scores	18.7 (1.0)	10.3 (1.2)	8.6 (1.3)	–8.4 (–11.1 to –5.6)	<.001	1.52	–10.1 (–13.1 to –7.1)	<.001	1.79
PHQ-9^d^ total scores	14.7 (0.8)	6.4 (1.0)	6.1 (0.9)	–8.3 (–10.3 to –6.2)	<.001	1.89	–8.6 (–10.6 to –6.6)	<.001	2.03
WSAS^e^ total scores	22.0 (1.6)	12.1 (1.4)	10.7 (1.6)	–9.8 (–13.7 to –6.0)	<.001	1.32	–11.2 (–15.2 to –7.3)	<.001	1.42
Q-LES-Q-SF^f^ % scores	41.7 (2.3)	63.7 (2.8)	65.3 (2.7)	22.0 (14.5 to 29.6)	<.001	1.73	23.6 (16.7 to 30.5)	<.001	1.92

^a^LSM = least squares mean.

^b^Within-group effect sizes were calculated as Hedges *g_ave_* for differences from baseline to week 8 or 20, respectively, using raw means for completers only.

^c^HAM-D: Hamilton Depression Rating Scale (score range 0 to 52, where higher scores indicate greater depression severity).

^d^PHQ-9: Patient Health Questionnaire-9 item (score range 0 to 27, where higher scores indicate greater depression severity).

^e^WSAS: Work and Social Adjustment Scale (score range 0 to 40, where higher scores mean higher functional impairment).

^f^Q-LES-Q-SF: Quality of Life Enjoyment and Satisfaction Questionnaire-Short Form (percent score range 0% to 100%, where higher scores correspond to greater ratings of quality of life).

#### Adverse Events and Medication Changes

Overall, 17 out of 28 participants reported a total of 33 adverse events during the 8-week treatment phase of the trial in categories such as psychiatric symptoms (n=21, 64%; eg, increased suicidal ideation, depression, or anxiety, sleep difficulties, and emotional distress), infections (n=6, 18%; eg, COVID-19, shingles, and illness), physical injuries (n=3, 9%), and other (n=3, 9%). All adverse events were identified as either mild (new event that did not interfere with activities of daily living; 25/33, 75.8%) or moderate (new event that posed some interference or required intervention to prevent interference; 8/33, 24.2%). No serious adverse events occurred in this trial. By assessing how likely it was that reported adverse events were related to treatment, most events were found to be definitely unrelated (19/33, 57.6%), followed by unlikely to be related (5/33, 15.2%) or possibly related (9/33, 27.3%). A waxing and waning course of MDD symptoms and suicidal ideation is common in MDD. However, there were no adverse events indicating significant clinical deterioration in the trial and no principal investigator–initiated withdrawals. Two participants changed psychotropic medications during the treatment phase: 1 participant increased the dosage of their medication for anxiety and 1 participant discontinued their medication for depression. There were no reported therapy changes during the treatment phase. In the 3-month follow-up phase of the trial, 7 participants reported an additional 10 adverse events in the categories of psychiatric symptoms (4/10, 40%), general disorders (2/10, 20%), and other (4/10, 40%), which were found to be definitely unrelated (5/10, 50%), unlikely to be related (1/10, 10%), and possibly related (4/10, 40%) to treatment. Also during the follow-up period, 5 participants changed psychotropic medications and 3 participants started individual therapy or counseling (non-CBT; for mood or anxiety, and traumatic event or PTSD).

## Discussion

### Principal Findings

In this study, we examined the feasibility, acceptability, and preliminary clinical impact of Mindset for Depression, an 8-week app-based CBT with therapist support. The results support Mindset for Depression as a viable treatment option for individuals with moderate to severe MDD. Treatment was feasible to deliver in a setting and acceptable to patients who varied widely in age, severity of the symptoms, and other clinical and demographic dimensions, as indicated by high retention rates (27/29, 93%), favorable satisfaction ratings (CSQ), and positive user feedback (uMARS). The results also showed that the treatment was efficacious. There was a significant reduction in clinician-rated (HAM-D) depression severity with a large effect size as well as significant improvement in functioning and quality of life. After just 8 weeks, about half of the participants were rated as treatment responders and a third were in remission, and these changes were maintained throughout the 3-month follow-up. These results are similar to face-to-face psychotherapy [54] and comparable to guided internet-delivered CBTs, notable given the short treatment duration, younger age, and relatively higher severity of the sample, all of which are associated with lower odds of response and remission in digital treatment [55].

Encouragingly, app ratings were above average for mental health apps [56]. Compared to other mHealth for depression or anxiety [57-60] and treatment-as-usual for depression [61], overall treatment satisfaction scores (CSQ) were also excellent. This was achieved despite clinician time that was below typical courses of CBT for depression (8 sessions averaging 24 minutes vs upwards of 20 sessions lasting on average 45-50 minutes) [62]. Indeed, much of the time-consuming didactic content (eg, psychoeducation about the CBT model) was administered by the app through readings, videos, and practice questions, conserving clinician time for more personalized skills’ review, practice, and tailoring and addressing risk issues. App usage data were excellent, with most participants reaching the final step by the end of 8 weeks as intended and reporting regularly practicing skills on or off the app each week. Moreover, participants rarely used the messaging function between sessions (eg, seeking additional clarification or encouragement), which would be unbillable clinician time. In this way, Mindset for Depression has the potential to improve the reach of CBT therapists and hopefully reduce treatment gaps, particularly for underserved communities [63,64].

These results are important because the high cost and limited availability of trained clinicians are major barriers to the dissemination of traditional psychotherapy [65]. Our findings add to an emerging literature demonstrating the potential of guided smartphone-based CBT to mitigate these challenges. However, although numerous seemingly efficacious therapist-supported digital treatments have been created for depression [10], few are available outside of research settings or integrated into a health care system [25]. Created in collaboration with an industry partner to accelerate the dissemination pipeline and allow for ongoing technical maintenance and improvements, Mindset for Depression is commercially available and poised to be truly scalable and successful in real clinical settings. Setting it apart, Mindset for Depression was collaboratively developed with a design team, clinicians, and people with lived experience as well as rigorously applied user interface and experience best practices for mobile platforms. Critically, “users” in the user-centered design process included both patients and clinicians. This approach aligns clinical and engagement incentives so that one is not delivered at the expense of the other and yields an easy to use, streamlined, and effective treatment program. Concretely, this translated to pacing content (delivering or unlocking intervention components in a stepwise manner to encourage practice and mastery of concepts and skills that build on one another), shorter activity lengths, creating a professional and approachable tone, and inclusion of feedback loops. As standardized content is delivered via the app in each step, supporting clinicians are able to prioritize personalizing treatment and use their specialized skillsets, such as addressing unique barriers to motivation, engagement, or response. Critical next steps would be to directly evaluate the program and its readiness to scale in larger scale effectiveness trials and real-world settings.

The results also provide important guidance for improving the program. First, participant feedback (eg, uMARS engagement subscale) indicates that increased customization and interactivity could improve the app’s appeal. This is consistent with the larger literature showing user preferences for apps with such features and negative reactions to apps whose content is repetitive and not personally relevant [66]. Although most participants shared positive views of the Mindset app, including indicating that they would recommend the app to friends, there was a mixed response regarding their willingness to pay for the Mindset app. It is unclear to what extent this reflects (1) that this question was asked after treatment and thus patients no longer felt the need to use the app; patients were meant to complete all therapeutic content within the 8-week treatment period; (2) a gap between what patients find beneficial and what they are willing to pay for; other studies have similarly found a reluctance to pay for mental health apps [66,67]; or (3) whether the app and concurrent therapist support were experienced as critically linked, and thus the app alone was not as valued. Indeed, half of participants indicated weekly brief sessions were the exact right amount of therapist contact and only 2 would have preferred less contact. Resolving this question will be necessary for developing a commercially sustainable implementation plan. Moreover, future iterations would benefit from broadening outcomes of interest. For example, beyond reducing symptoms of depression or other mental health concerns, an optimal intervention would also foster positive emotions and thriving and perhaps target common comorbidities, such as sleep difficulties or substance use. These outcomes should be captured in future studies and additional treatment components integrated as appropriate.

### Limitations

The study has limitations that should be considered. First, the study has the inherent limitation of an open trial. Without a control group, we cannot conclusively determine that the treatment causes improvements in symptoms. Future controlled large-scale trials are needed. Second, the patient sample was self-selected, recruitment platforms were diverse, and our study therapists were trained in the use of digital therapeutics. Thus, patient and clinician stakeholders might have been biased toward individuals who are motivated by app-based therapy. Future research in real-world clinical settings is warranted. Third, although the large proportion of White women in the sample is consistent with past work and higher MDD prevalence and rate of treatment seeking in women [10], greater representation of patients with other racial and gender identities would strengthen our conclusions and ongoing treatment improvements. Fourth, we had adequate power to detect moderate to large treatment effects; a larger replication is needed to explore moderators and mediators, which are important for tiered care models. Finally, a longer follow-up period and health economics metrics would be required to see the full time and cost-savings potential of Mindset for Depression.

### Conclusions

Mindset for Depression offers flexible app-led psychoeducation, skills practice, and support to patients with complementary clinician guidance to promote sustained engagement, monitor safety, and tailor treatment further to individual patient needs. The findings show that Mindset for Depression is a feasible, acceptable, and efficacious tool for adults with MDD. The hope is that such a program could be one cost-effective solution to barriers to psychotherapy dissemination and significantly increase access to evidence-based care. Although these initial results are very promising, more work remains to personalize the amount of therapist support and dose of treatment individuals receive to optimize treatment and increase rates of response and remission. The next steps include testing Mindset for Depression in a fully powered randomized controlled trial as well as the real-world clinical settings in which it is deployed.

## Appendix

Multimedia Appendix 1 Mindset Therapist Adherence Scale.

## Abbreviations

CBT: cognitive behavioral therapy
CEQ: Credibility/Expectancy Questionnaire
CSQ: Client Satisfaction Questionnaire
DSM-5: Diagnostic and Statistical Manual of Mental Disorders, Fifth Edition
HAM-D: Hamilton Depression Rating Scale
HIPAA: Health Insurance Portability and Accountability Act
MDD: major depressive disorder
PHQ-9: Patient Health Questionnaire-9
Q-LES-Q-SF: The Quality of Life Enjoyment and Satisfaction Questionnaire-Short Form
SMART: specific, measurable, achievable, relevant, and time-bound
uMARS: Mobile Application Rating Scale User Version
WSAS: Work and Social Adjustment Scale

## References

[1] (2013). Diagnostic and Statistical Manual of Mental Disorders, 5th Edition.

[2] (2020). 2020 National Survey on Drug Use and Health (NSDUH) releases. Substance Abuse and Mental Health Services Administration.

[3] Ettman CK, Cohen GH, Abdalla SM, Sampson L, Trinquart L, Castrucci BC, Bork RH, Clark MA, Wilson I, Vivier PM, Galea S (2022). Persistent depressive symptoms during COVID-19: a national, population-representative, longitudinal study of U.S. adults. Lancet Reg Health Am.

[4] Friedrich MJ (2017). Depression is the leading cause of disability around the world. JAMA.

[5] Greenberg PE, Fournier AA, Sisitsky T, Simes M, Berman R, Koenigsberg SH, Kessler RC (2021). The economic burden of adults with major depressive disorder in the United States (2010 and 2018). Pharmacoeconomics.

[6] Moitra M, Santomauro D, Collins PY, Vos T, Whiteford H, Saxena S, Ferrari AJ (2022). The global gap in treatment coverage for major depressive disorder in 84 countries from 2000-2019: a systematic review and Bayesian meta-regression analysis. PLoS Med.

[7] López-López JA, Davies SR, Caldwell DM, Churchill R, Peters TJ, Tallon D, Dawson S, Wu Q, Li J, Taylor A, Lewis G, Kessler DS, Wiles N, Welton NJ (2019). The process and delivery of CBT for depression in adults: a systematic review and network meta-analysis. Psychol Med.

[8] Andrade LH, Alonso J, Mneimneh Z, Wells JE, Al-Hamzawi A, Borges G, Bromet E, Bruffaerts R, de Girolamo G, de Graaf R, Florescu S, Gureje O, Hinkov HR, Hu C, Huang Y, Hwang I, Jin R, Karam EG, Kovess-Masfety V, Levinson D, Matschinger H, O'Neill S, Posada-Villa J, Sagar R, Sampson NA, Sasu C, Stein DJ, Takeshima T, Viana MC, Xavier M, Kessler RC (2014). Barriers to mental health treatment: results from the WHO World Mental Health surveys. Psychol Med.

[9] Webb CA, Rosso IM, Rauch SL (2017). Internet-based cognitive-behavioral therapy for depression: current progress and future directions. Harv Rev Psychiatry.

[10] Børtveit L, Dechsling A, Sütterlin S, Nordgreen T, Nordahl-Hansen A (2022). Guided internet-delivered treatment for depression: scoping review. JMIR Ment Health.

[11] Torous J, Levin ME, Ahern DK, Oser ML (2017). Cognitive behavioral mobile applications: clinical studies, marketplace overview, and research agenda. Cogn Behav Pract.

[12] Linardon J, Cuijpers P, Carlbring P, Messer M, Fuller-Tyszkiewicz M (2019). The efficacy of app-supported smartphone interventions for mental health problems: a meta-analysis of randomized controlled trials. World Psychiatry.

[13] Mohr DC, Azocar F, Bertagnolli A, Choudhury T, Chrisp P, Frank R, Harbin H, Histon T, Kaysen D, Nebeker C, Richards D, Schueller SM, Titov N, Torous J, Areán PA (2021). Banbury forum consensus statement on the path forward for digital mental health treatment. Psychiatr Serv.

[14] Wasil AR, Gillespie S, Shingleton R, Wilks CR, Weisz JR (2020). Examining the reach of smartphone apps for depression and anxiety. Am J Psychiatry.

[15] Lattie EG, Schueller SM, Sargent E, Stiles-Shields C, Tomasino KN, Corden ME, Begale M, Karr CJ, Mohr DC (2016). Uptake and usage of Intellicare: a publicly available suite of mental health and well-being apps. Internet Interv.

[16] Baumel A, Muench F, Edan S, Kane JM (2019). Objective user engagement with mental health apps: systematic search and panel-based usage analysis. J Med Internet Res.

[17] Mohr DC, Cuijpers P, Lehman K (2011). Supportive accountability: a model for providing human support to enhance adherence to eHealth interventions. J Med Internet Res.

[18] Mohr DC, Schueller SM, Tomasino KN, Kaiser SM, Alam N, Karr C, Vergara JL, Gray EL, Kwasny MJ, Lattie EG (2019). Comparison of the effects of coaching and receipt of app recommendations on depression, anxiety, and engagement in the IntelliCare platform: factorial randomized controlled trial. J Med Internet Res.

[19] Torous J, Nicholas J, Larsen ME, Firth J, Christensen H (2018). Clinical review of user engagement with mental health smartphone apps: evidence, theory and improvements. Evid Based Ment Health.

[20] Muñoz RF (2017). The efficiency model of support and the creation of digital apothecaries. Clin Psychol (New York).

[21] Andersson G, Topooco N, Havik O, Nordgreen T (2016). Internet-supported versus face-to-face cognitive behavior therapy for depression. Expert Rev Neurother.

[22] Andersson G, Cuijpers P, Carlbring P, Riper H, Hedman E (2014). Guided internet-based vs. face-to-face cognitive behavior therapy for psychiatric and somatic disorders: a systematic review and meta-analysis. World Psychiatry.

[23] Carlbring P, Andersson G, Cuijpers P, Riper H, Hedman-Lagerlöf E (2018). Internet-based vs. face-to-face cognitive behavior therapy for psychiatric and somatic disorders: an updated systematic review and meta-analysis. Cogn Behav Ther.

[24] Shechter A, Diaz F, Moise N, Anstey DE, Ye S, Agarwal S, Birk JL, Brodie D, Cannone DE, Chang B, Claassen J, Cornelius T, Derby L, Dong M, Givens RC, Hochman B, Homma S, Kronish IM, Lee SAJ, Manzano W, Mayer LES, McMurry CL, Moitra V, Pham P, Rabbani L, Rivera RR, Schwartz A, Schwartz JE, Shapiro PA, Shaw K, Sullivan AM, Vose C, Wasson L, Edmondson D, Abdalla M (2020). Psychological distress, coping behaviors, and preferences for support among New York healthcare workers during the COVID-19 pandemic. Gen Hosp Psychiatry.

[25] Bernstein EE, Weingarden H, Wolfe EC, Hall MD, Snorrason I, Wilhelm S (2022). Human support in app-based cognitive behavioral therapies for emotional disorders: scoping review. J Med Internet Res.

[26] Chang S, Gray L, Torous J (2023). Smartphone app engagement and clinical outcomes in a hybrid clinic. Psychiatry Res.

[27] Lakhtakia T, Torous J (2022). Current directions in digital interventions for mood and anxiety disorders. Curr Opin Psychiatry.

[28] Karyotaki E, Efthimiou O, Miguel C, Bermpohl FMG, Furukawa TA, Cuijpers P, Riper H, Patel V, Mira A, Gemmil AW, Yeung AS, Lange A, Williams AD, Mackinnon A, Geraedts A, van Straten A, Meyer B, Björkelund C, Knaevelsrud C, Beevers CG, Botella C, Strunk DR, Mohr DC, Ebert DD, Kessler D, Richards D, Littlewood E, Forsell E, Feng F, Wang F, Andersson G, Hadjistavropoulos H, Christensen H, Ezawa ID, Choi I, Rosso IM, Klein JP, Shumake J, Garcia-Campayo J, Milgrom J, Smith J, Montero-Marin J, Newby JM, Bretón-López J, Schneider J, Vernmark K, Bücker L, Sheeber LB, Warmerdam L, Farrer L, Heinrich M, Huibers MJH, Kivi M, Kraepelien M, Forand NR, Pugh N, Lindefors N, Lintvedt O, Zagorscak P, Carlbring P, Phillips R, Johansson R, Kessler RC, Brabyn S, Perini S, Rauch SL, Gilbody S, Moritz S, Berger T, Pop V, Kaldo V, Spek V, Forsell Y, Individual Patient Data Meta-Analyses for Depression (IPDMA-DE) Collaboration (2021). Internet-based cognitive behavioral therapy for depression: a systematic review and individual patient data network meta-analysis. JAMA Psychiatry.

[29] Depression in adults: treatment and management [NICE guideline NG222]. National Institute for Health and Care Excellence.

[30] Posner K, Brent D, Lucas C, Gould M, Stanley B, Brown G, Fisher P, Zelazny J, Burke J, Mann J (2008). Columbia-Suicide Severity Rating Scale (C-SSRS). The Columbia Lighthouse Project.

[31] Forde F, Frame M, Hanlon P, MacLean G, Nolan D, Shajahan P, Troy E (2005). Optimum number of sessions for depression and anxiety. Nurs Times.

[32] Mohr DC, Weingardt KR, Reddy M, Schueller SM (2017). Three problems with current digital mental health research . . . and three things we can do about them. Psychiatr Serv.

[33] Ciharova M, Furukawa TA, Efthimiou O, Karyotaki E, Miguel C, Noma H, Cipriani A, Riper H, Cuijpers P (2021). Cognitive restructuring, behavioral activation and cognitive-behavioral therapy in the treatment of adult depression: a network meta-analysis. J Consult Clin Psychol.

[34] Sauer-Zavala S, Bentley KH, Steele SJ, Tirpak JW, Ametaj AA, Nauphal M, Cardona N, Wang M, Farchione TJ, Barlow DH (2020). Treating depressive disorders with the Unified Protocol: a preliminary randomized evaluation. J Affect Disord.

[35] Beck AT (1979). Cognitive Therapy of Depression.

[36] Bentley KH, Bernstein EE, Wallace B, Mischoulon D (2021). Treatment for anxiety and comorbid depressive disorders: transdiagnostic cognitive-behavioral strategies. Psychiatr Ann.

[37] Dimidjian S, Martell CR, Herman-Dunn R, Hubley S, Barlow DH (2014). Behavioral activation for depression. Clinical Handbook of Psychological Disorders: A Step-by-Step Treatment Manual, 5th Edition.

[38] Sturmey P (2009). Behavioral activation is an evidence-based treatment for depression. Behav Modif.

[39] MacKenzie MB, Kocovski NL (2016). Mindfulness-based cognitive therapy for depression: trends and developments. Psychol Res Behav Manag.

[40] Akçayır G, Akçayır M (2018). The flipped classroom: a review of its advantages and challenges. Comput Educ.

[41] Sheehan DV, Lecrubier Y, Sheehan KH, Amorim P, Janavs J, Weiller E, Hergueta T, Baker R, Dunbar GC (1998). The Mini-International Neuropsychiatric Interview (M.I.N.I.): the development and validation of a structured diagnostic psychiatric interview for DSM-IV and ICD-10. J Clin Psychiatry.

[42] Devilly GJ, Borkovec TD (2000). Psychometric properties of the credibility/expectancy questionnaire. J Behav Ther Exp Psychiatry.

[43] Stoyanov SR, Hides L, Kavanagh DJ, Wilson H (2016). Development and validation of the user Version of the Mobile Application Rating Scale (uMARS). JMIR Mhealth Uhealth.

[44] Attkisson CC, Zwick R (1982). The client satisfaction questionnaire. Psychometric properties and correlations with service utilization and psychotherapy outcome. Eval Program Plann.

[45] Hamilton M (1960). A rating scale for depression. J Neurol Neurosurg Psychiatry.

[46] Rosenblat JD, Lee Y, McIntyre RS (2018). The effect of pharmacogenomic testing on response and remission rates in the acute treatment of major depressive disorder: a meta-analysis. J Affect Disord.

[47] Nierenberg AA, DeCecco LM (2001). Definitions of antidepressant treatment response, remission, nonresponse, partial response, and other relevant outcomes: a focus on treatment-resistant depression. J Clin Psychiatry.

[48] Frank E, Prien RF, Jarrett RB, Keller MB, Kupfer DJ, Lavori PW, Rush AJ, Weissman MM (1991). Conceptualization and rationale for consensus definitions of terms in major depressive disorder. Remission, recovery, relapse, and recurrence. Arch Gen Psychiatry.

[49] Mundt JC, Marks IM, Shear MK, Greist JH (2002). The work and social adjustment scale: a simple measure of impairment in functioning. Br J Psychiatry.

[50] Endicott J, Nee J, Harrison W, Blumenthal R (1993). Quality of life enjoyment and satisfaction questionnaire: a new measure. Psychopharmacol Bull.

[51] Spitzer RL, Kroenke K, Williams JB (1999). Validation and utility of a self-report version of PRIME-MD: the PHQ primary care study. Primary care evaluation of mental disorders. Patient health questionnaire. JAMA.

[52] Cuijpers P, Cristea IA, Karyotaki E, Reijnders M, Huibers MJH (2016). How effective are cognitive behavior therapies for major depression and anxiety disorders? A meta-analytic update of the evidence. World Psychiatry.

[53] Lakens D (2013). Calculating and reporting effect sizes to facilitate cumulative science: a practical primer for t-tests and ANOVAs. Front Psychol.

[54] Cuijpers P, Karyotaki E, Weitz E, Andersson G, Hollon SD, van Straten A (2014). The effects of psychotherapies for major depression in adults on remission, recovery and improvement: a meta-analysis. J Affect Disord.

[55] Karyotaki E, Ebert DD, Donkin L, Riper H, Twisk J, Burger S, Rozental A, Lange A, Williams AD, Zarski AC, Geraedts A, van Straten A, Kleiboer A, Meyer B, Ince BBÜ, Buntrock C, Lehr D, Snoek FJ, Andrews G, Andersson G, Choi I, Ruwaard J, Klein JP, Newby JM, Schröder J, Laferton JA, Van Bastelaar K, Imamura K, Vernmark K, Boß L, Sheeber LB, Kivi M, Berking M, Titov N, Carlbring P, Johansson R, Kenter R, Perini S, Moritz S, Nobis S, Berger T, Kaldo V, Forsell Y, Lindefors N, Kraepelien M, Björkelund C, Kawakami N, Cuijpers P (2018). Do guided internet-based interventions result in clinically relevant changes for patients with depression? An individual participant data meta-analysis. Clin Psychol Rev.

[56] Hudson G, Negbenose E, Neary M, Jansli SM, Schueller SM, Wykes T, Jilka S (2022). Comparing professional and consumer ratings of mental health apps: mixed methods study. JMIR Form Res.

[57] Schlicker S, Baumeister H, Buntrock C, Sander L, Paganini S, Lin J, Berking M, Lehr D, Ebert DD (2020). A web- and mobile-based intervention for comorbid, recurrent depression in patients with chronic back pain on sick leave (Get.Back): pilot randomized controlled trial on feasibility, user satisfaction, and effectiveness. JMIR Ment Health.

[58] Netter AL, Beintner I, Brakemeier EL (2022). Adding an app-based intervention to the cognitive behavioral analysis system of psychotherapy in routine outpatient psychotherapy treatment: proof-of-concept study. JMIR Form Res.

[59] Sharma G, Schlosser L, Jones BDM, Blumberger DM, Gratzer D, Husain MO, Mulsant BH, Rappaport L, Stergiopoulos V, Husain MI (2022). Brief app-based cognitive behavioral therapy for anxiety symptoms in psychiatric inpatients: feasibility randomized controlled trial. JMIR Form Res.

[60] Gotthardt M, Striegl J, Loitsch C, Weber G, Miesenberger K, Kouroupetroglou G, Mavrou K, Manduchi R, Rodriguez MC, Penáz P (2022). Voice assistant-based CBT for depression in students: effects of empathy-driven dialog management. Computers Helping People with Special Needs. ICCHP-AAATE 2022. Lecture Notes in Computer Science, Vol 13341.

[61] Richards DA, Hill JJ, Gask L, Lovell K, Chew-Graham C, Bower P, Cape J, Pilling S, Araya R, Kessler D, Bland JM, Green C, Gilbody S, Lewis G, Manning C, Hughes-Morley A, Barkham M (2013). Clinical effectiveness of collaborative care for depression in UK primary care (CADET): cluster randomised controlled trial. BMJ.

[62] Cuijpers P, Berking M, Andersson G, Quigley L, Kleiboer A, Dobson KS (2013). A meta-analysis of cognitive-behavioural therapy for adult depression, alone and in comparison with other treatments. Can J Psychiatry.

[63] Ramos G, Chavira DA (2022). Use of technology to provide mental health care for racial and ethnic minorities: evidence, promise, and challenges. Cogn Behav Pract.

[64] Bae H, Shin H, Ji HG, Kwon JS, Kim H, Hur JW (2023). App-based interventions for moderate to severe depression: a systematic review and meta-analysis. JAMA Netw Open.

[65] Cavanagh K (2014). Geographic inequity in the availability of cognitive behavioural therapy in England and Wales: a 10-year update. Behav Cogn Psychother.

[66] Balaskas A, Schueller SM, Cox AL, Rashleigh C, Doherty G (2023). Examining young adults daily perspectives on usage of anxiety apps: a user study. PLOS Digit Health.

[67] Bakker D, Kazantzis N, Rickwood D, Rickard N (2018). Development and pilot evaluation of smartphone-delivered cognitive behavior therapy strategies for mood- and anxiety-related problems: MoodMission. Cogn Behav Pract.

