# Supplementary material for: Feasibility, Acceptability, and Preliminary Efficacy of a Smartphone App–Led Cognitive Behavioral Therapy for Depression Under Therapist Supervision: Open Trial
Source: JMIR Ment Health. 2024 Apr 9;11:e53998. doi: 10.2196/53998 (PMC11040445; doi:10.2196/53998)
Supplement: Multimedia Appendix 1 [file mental_v11i1e53998_app1.docx]

**Supplemental Materials**

**Mindset Therapist Adherence Scale**

**Instructions:** *Use this scale to rate adherence to instructions in the treatment manual. For each adherence item below, rate the extent to which the therapist completed it, using the following scale:*

1=Not at all 2=Very Little 3=Little 4= Somewhat 5=Moderately 6=Mostly 7=Completely N/A=Not applicable

*NOTE: Use bulleted items as a general guideline for what the therapist should have done in the session. However, as those are very detailed, your rating should capture only what is marked in bold font.*

**SESSION 1**

| 1. **Introduction**   *Did the therapist …* | 1 2 3 4 5 6 7  N/A |
| --- | --- |
| - Introduce self? - Ask the patient to describe themselves (current context)? - Review limits of confidentiality and emergency procedures? |  |
| 1. **Assessment**   *Did the therapist…* | 1 2 3 4 5 6 7  N/A |
| - - Provide an overview from the patient’s baseline assessment?   - Ask for feedback from the patient?   - Monitor and address suicidal ideation? |  |
| 1. **Orientation to Treatment**   *Did the therapist do one or more of the following…* | 1 2 3 4 5 6 7  N/A |
| - - Explain the purpose, features, expectations of the app treatment?   - Overview of the messaging features?   - Review the importance of practice of the exercises on and off the app?   - Review treatment goals?   - Enhance motivation (if applicable)? |  |
| 1. **Therapeutic Content**   *Did the therapist do one or more of the following…* | 1 2 3 4 5 6 7  N/A |
| - - Ask the patient to describe what they have encountered thus far in the app?   - Review content and answer any questions?   - Complete in-session practice? |  |
| 1. **Homework**   *Did the therapist do one or more of the following…* | 1 2 3 4 5 6 7  N/A |
| - - Encourage the patient to review and practice previous skills (if applicable)?   - Collaboratively determine homework for the next week based on what is meant to be practiced in the app?   - Review the importance of practice of the exercises on and off the app?   - Emphasize goals that are specific (to the app and the patient) and reasonable? |  |
| 1. **FORBIDDEN:**   *Check to make sure the therapist did NOT…*   - Teach therapeutic concepts or skill not included in the Mindset app   *Included modules: CBT model of depression, cognitive restructuring, behavioral activation, mindfulness/grounding* | 1 2 3 4 5 6 7  N/A |
| 1. **Overall Adherence Rating**   *Please rate the therapist’s overall adherence.* | 1 2 3 4 5 6 7 |

**SESSIONS 2-7**

| 1. **Symptom and Safety Check**   *Did the therapist …* | 1 2 3 4 5 6 7  N/A |
| --- | --- |
| - Review weekly questionnaires? - Monitor and address suicidal ideation? |  |
| 1. **Homework Review**   *Did the therapist do one or more of the following…* | 1 2 3 4 5 6 7  N/A |
| - - Check to see if the patient completed the assigned practice?   - Address any questions or concerns?   - Celebrate the successes, such as what the patient completed or what went well?   - Problem-solve any barriers to homework completion? |  |
| 1. **Review of New Content**   *Did the therapist do one or more of the following…* | 1 2 3 4 5 6 7  N/A |
| - - Ask the patient to describe what they have learned and practiced in and out of the app?   - Review content and answer any questions?   - Complete in-session practice?   - Encourage the patient to review and practice previous skills? |  |
| 1. **Homework**   *Did the therapist do one or more of the following…* | 1 2 3 4 5 6 7  N/A |
| - - Collaboratively determine homework for the next week based on what is meant to be practiced in the app?   - Review the importance of practice?   - Emphasize goals that are specific (to the app and the patient) and reasonable?   - Encourage/help the patient to get back on track if they were delayed? |  |
| 1. **FORBIDDEN:**   *Check to make sure the therapist did NOT…*   - Teach therapeutic concepts or skill not included in the app?   *Included modules: CBT model of depression, cognitive restructuring, behavioral activation, mindfulness/grounding* | 1 2 3 4 5 6 7  N/A |
| 1. **Overall Adherence Rating**   *Please rate the therapist’s overall adherence.* | 1 2 3 4 5 6 7 |

**SESSION 8**

| 1. **Symptom and Safety Check**   *Did the therapist …* | 1 2 3 4 5 6 7  N/A |
| --- | --- |
| - Review weekly questionnaires? - Monitor and address suicidal ideation? |  |
| 1. **Homework Review**   *Did the therapist do one or more of the following…* | 1 2 3 4 5 6 7  N/A |
| - - Check to see if the patient completed the assigned practice?   - Address any questions or concerns?   - Celebrate the successes, such as what the patient completed or what went well?   - Problem-solve any barriers to homework completion? |  |
| 1. **Relapse Prevention**   *Did the therapist do one or more of the following…* | 1 2 3 4 5 6 7  N/A |
| - - Discuss possible symptom recurrence?   - Discuss strategies to handle lapses and setbacks? |  |
| 1. **Review Course of Treatment**   *Did the therapist do one or more of the following…* | 1 2 3 4 5 6 7  N/A |
| - - Ask the patient to summarize skills learned in treatment?   - Address questions or concerns?   - Celebrate treatment gains and reference initial treatment goals?   - Discuss patient’s new or ongoing goals? |  |
| 1. **FORBIDDEN:**   *Check to make sure the therapist did NOT…*   - Teach therapeutic concepts or skill not included in the app?   *Included modules: CBT model of depression, cognitive restructuring, behavioral activation, mindfulness/grounding* | 1 2 3 4 5 6 7  N/A |
| 1. **Overall Adherence Rating**   *Please rate the therapist’s overall adherence.* | 1 2 3 4 5 6 7 |
